# *De Novo* Assembly and Characterization of the Fruit Transcriptome of Chinese Jujube (*Ziziphus jujuba* Mill.) Using 454 Pyrosequencing and the Development of Novel Tri-Nucleotide SSR Markers

**DOI:** 10.1371/journal.pone.0106438

**Published:** 2014-09-03

**Authors:** Yingyue Li, Chaoqun Xu, Xinggu Lin, Binbin Cui, Rongling Wu, Xiaoming Pang

**Affiliations:** 1 National Engineering Laboratory for Tree Breeding, Key Laboratory of Genetics and Breeding in Forest Trees and Ornamental Plants, Ministry of Education, College of Biological Sciences and Biotechnology, Beijing Forestry University, Beijing, China; 2 Center for Computational Biology, Beijing Forestry University, Beijing, China; Key Laboratory of Horticultural Plant Biology (MOE), China

## Abstract

Chinese jujube (*Ziziphus jujuba* Mill.) is an economically important deciduous tree that has high therapeutic value and health benefits. However, a lack of sequence data and molecular markers have constrained genetic and breeding studies for better fruit quality and other traits in Chinese jujube. In this study, two combined cDNA libraries of ‘Dongzao’ fruit representing the early and late stages of fruit development were constructed and sequenced on the 454 GS FLX Titanium platform. In total, 1,124,197 reads were generated and then *de novo* assembled into 97,479 unigenes. A total of 52,938 unigenes were homologous to genes in the NCBI non-redundant sequence database. A total of 33,123 unigenes were assigned to one or more Gene Ontology terms, and 16,693 unigenes were classified into 319 Kyoto Encyclopedia of Genes and Genomes pathways. The results showed that the Smirnoff-Wheeler pathway was the main pathway for the biosynthesis of ascorbic acid in Chinese jujube. The number of differentially expressed genes between the two stages of fruit development was 1,764, among which 974 and 790 genes were up-regulated and down-regulated, respectively. Furthermore, 9,893 sequences were identified containing SSRs. 93 primer pairs designed from the sequences with a tri-nucleotide repeat showed successful PCR amplification and could be validated in Chinese jujube accessions and *Z. mauritiana Lam* and *Z. acidojujuba* as well, of which 71 primer pairs were polymorphic. The obtained transcriptome provides a most comprehensive resource currently available for gene discovery and the development of functional markers in *Z. jujuba*. The newly developed microsatellite markers could be used in applications such as genetic linkage analysis and association studies, diversity analysis, and marker-assisted selection in Chinese jujube and related species.

## Introduction

*Ziziphus* species of the Rhamnaceae, the buckthorn family, are widespread in both hemispheres [1]. Chinese jujube (*Ziziphus jujuba* M.) and Indian jujube (*Z. mauritiana* Lam.) are two species that have considerable horticultural importance [1]. Approximately 170 other species exist worldwide, some of local importance. Chinese jujube (also known as Chinese date) is an important deciduous fruit tree that is grown in temperate and subtropical areas and is one of the most important fruit trees in China, where it has been cultivated and utilized for more than 4,000 years [2]. Due to its wider adaptability under adverse soil and climatic conditions and due to the multiple uses of jujube fruit, Chinese jujube is ranked first in dried fruit production in China, with an average annual production of 3.5 million tons (dried weight) [3]. Chinese jujube has a diploid genome (2n  =  2×  =  24) that is estimated to be about 430 Mbp [4]. Two triploid cultivars, ‘Zanghuangdazao’ and ‘Pingguozao,’ are also found in nature [[5]–[6], Pang et al, unpublished data]. The jujube fruit is an edible oval drupe, 1.5 to 6.0 centimeters long [1]. The fruit is rich in nutritive substances, including potassium, phosphorus, calcium and manganese, which are the major mineral components, as well as iron, sodium, zinc, copper and biologically active components, including vitamin C, phenolics, flavonoids, triterpenic acids, cyclic adenosine monophosphate (cAMP) and polysaccharides [7]–[8]. Furthermore, the fruits have also been used in traditional Chinese medicine to treat anorexia, fatigue, and loose stools in deficiency syndromes of the spleen and of hysteria in women [9]. In recent years, additional health benefits of Chinese jujube fruit have been reported, including anticancer, anti-inflammatory, anti-obesity, immunostimulating, antioxidant, hepatoprotective, and gastrointestinal protective activities and roles in the inhibition of foam cell formation in macrophages [9]. Chinese jujube can be consumed fresh, dehydrated, canned or processed into candy, jam, juice, wine, syrup or vinegar [10].

The nutritional and sensorial attributes of the fruit are determined throughout the successive phases of fruit development, which includes cell division and the expansion of the ovary tissues in a highly coordinated, complex genetically programmed process that involves a series of physiological, biochemical and organoleptic changes [11]–[13]. The continuing development of genomics tools for important fruit crops has accelerated research in fruit development. Many genes regulating fruit development, such as *fw* 2.2 and *SUN*, have been identified, and the underlying mechanisms of fruit development have been extensively studied in several species, such as tomato, strawberry and apple [14]. A wide variety of Chinese jujube cultivars are grown in China that differ in their fruit size, shape, texture, taste and nutritional content [1], [15]. However, few studies have addressed the gene expression patterns in the fruit development of Chinese jujube. Recently, Liu et al. reported a fruit cDNA library with 965 unigenes and successfully developed 119 gene-derived SSR markers [16]. Lin et al. obtained 216 genes that are related to fruit softening using suppression subtractive hybridization (SSH) technology [17]. A lack of sequence data has constrained genetic and breeding studies for better fruit quality and other traits in Chinese jujube. Therefore, an improvement of our knowledge of the gene composition and expression is essential to investigate the molecular basis of fruit development in Chinese jujube.

Molecular markers represent one of the most powerful tools in the analysis of plant genomes and in the association of heritable phenotypic traits with underlying genetic variation [18]. During the past three decades, many different molecular markers have been developed [19]. Among these markers, simple sequence repeats (SSRs) and single nucleotide polymorphisms (SNPs) have been the predominating markers that are utilized in modern plant genetic analysis and marker-assisted breeding [19]–[20]. SNPs are the most abundant genetic markers in the genome [21]; however, sequence availability is the limiting factor for the discovery of SNPs in less-studied plant species. SSRs, also known as microsatellites, have many advantages, including high abundance, codominant inheritance, hypervariability and extensive genomic coverage [22]. SSRs can be divided into genomic SSRs, which are derived from genomic sequences, and EST-SSRs, which are derived from expressed sequence tags. Compared with the genomic SSRs, EST-SSRs have several special advantages due to either a relatively higher transferability or a potential gene function [23], which have been evaluated in many studies [24]–[27]. Currently, only a few SSR and EST-SSR primers have been developed in Chinese jujube [28], [29] and sour jujube [30]. The lack of suitable mapping populations and larger high-throughput marker collection limits gene isolation and breeding in Chinese jujube. Therefore, the development of a set of reliable SSR markers is an urgent requirement for genetic and breeding studies in Chinese jujube.

Expressed sequence tag (EST) sequencing is a cost-effective and frequently used strategy for the efficient and rapid identification of novel genes and the development of molecular markers. The development of high-throughput next generation sequencing (NGS) technologies offers the ability to sequence and characterize the transcriptome cheaply and quickly [31]. Transcriptome sequencing has led a new revolution in biological applications, especially in efficiently and cost-effectively identifying simple sequence repeat (SSR) regions for massive microsatellite marker development [32], [33], particularly in those species without reference genome sequences [34], [35]. The NGS technology provides new opportunities for a more accurate and powerful transcriptome analysis of fruit development, among which Roche 454 sequencing produces longer reads that are especially suited for *de novo* transcriptome sequencing [31].

In this study, we performed transcriptome sequencing during the early and late stages of Chinese jujube fruit development using the Roche 454 GS FLX Titanium sequencing platform. The transcriptome was first *de novo* assembled and annotated. We also revealed the differentially expressed genes between the early and late fruit development stages. Furthermore, we mined EST-SSR markers from the sequences and successfully developed 93 SSR markers. The extensive transcriptome data are important for and helpful in understanding the molecular mechanism of fruit development in Chinese jujube; the SSR markers developed in this study will facilitate gene mapping, linkage map development and marker-assisted selective breeding in Chinese jujube.

## Results and Discussion

### 454 sequencing and assembly

Two combined cDNA libraries of the ‘Dongzao’ fruit, i.e., S1 representing the early stage and S2 representing the late stage of fruit development based on the fruit growth curve (Figure S1), were constructed using the SMART cDNA Construction Kit (Clontech, USA). Then, these libraries were each sequenced in a half-plate run on the 454 GS FLX Titanium platform. In total, 1,140,509 raw reads were obtained, with an average length of 358 bp, which is longer than that in the floral transcriptome of the related species *Z. celata* [36]. After preprocessing, 1,124,197 high quality reads were finally used for the assembly (Table 1). The total sequence output was 396,961,286 nt (Table 1). All of the high quality reads were deposited in the National Center for Biotechnology Information (NCBI) and can be accessed in the Short Read Archive (SRA) under the accession number SRR1231563.

**Table 1:** Summary of transcriptome sequencing of Chinese jujube fruit using 454 GS FLX Titanium.

	S1	S2	ALL
Raw bases	173,067,941	235,252,344	408,320,285
Raw reads	482,227	658,282	1,140,509
Average reads length	358.9	357.4	358
Aligned bases	166,476,294	230,484,992	396,961,286
Aligned reads	467,943	656,254	1,124,197
All bases in isotigs	10,103,139	13,831,543	21,682,461
Isotig number	9,957	10,293	20,689
Contig number	11,566	13,805	25,805
Singleton number	64,350	61,577	76,790
Unigene number	74,307	84,600	97,479

**Figure 1:**
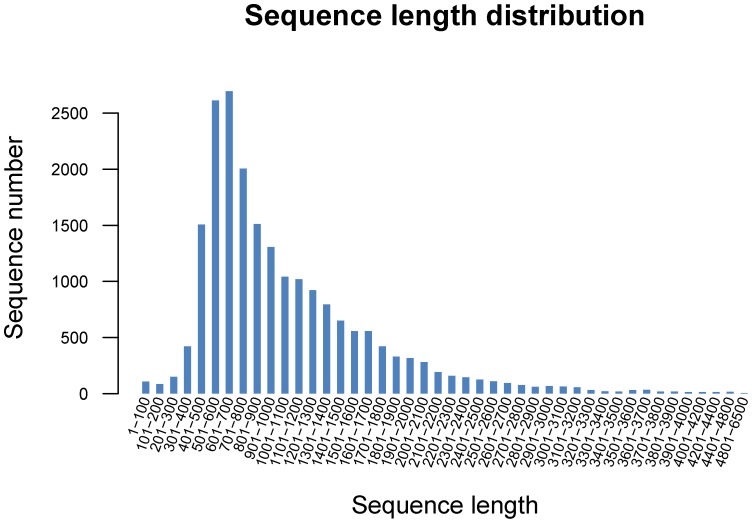
Sequence length distribution of all isotigs.

**Figure 2:**
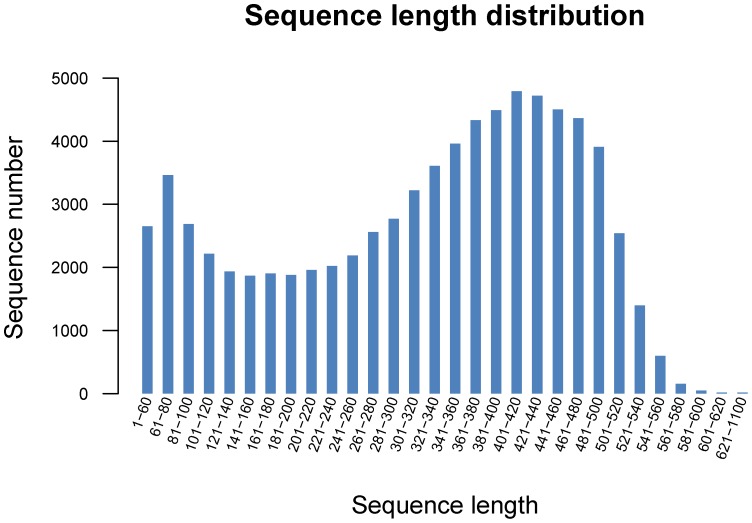
Sequence length distribution of all singletons.

**Figure 3:**
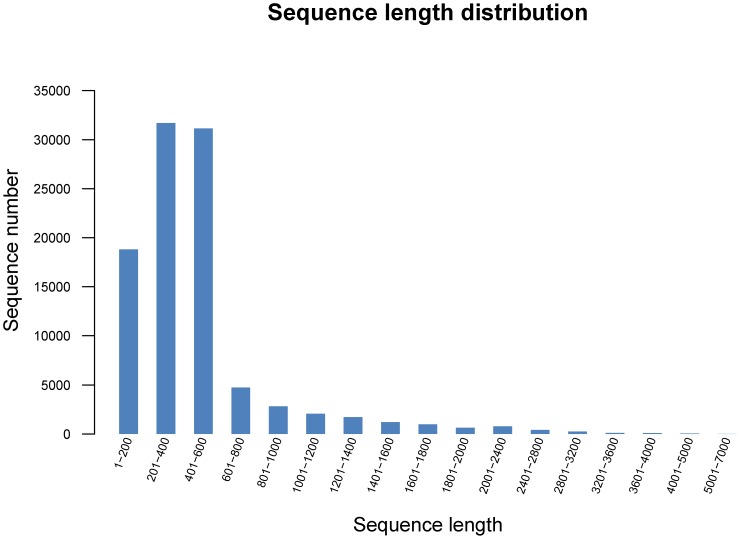
Assessment of assembled quality. The assembled quality of unigenes was assessed by the distribution of mapped reads within the assembled unigenes.

The GS *de novo* assembler software (Roche NEWBLER v2.3) was used to assemble the reads. Each putative isoform that was identified by Newbler is termed an isotig, and multiple isoforms for each gene are organized into isogroups, representing putative gene loci [37]. A singleton sequence is a sequence that is not assembled into contigs. For the S1 library, a total of 467,943 reads were assembled into 9,957 isotigs and 64,350 singletons for a total of 74,307 unigenes, whereas the S2 library produced 10,293 isotigs and 61,577 singletons for a total of 84,600 unigenes from 656,254 reads (Table 1). When the two libraries were analyzed together, we eventually obtained 97,479 unigenes, which consisted of 20,689 isotigs and 76,790 singletons. The singletons have a high possibility of representing genes that are expressed at low levels [38]. The large number of singleton sequences with BLAST matches indicates that the unassembled sequences provide an abundance of valuable sequence information and improve the overall transcriptome coverage breadth [36]. The sequence length distribution of all of the isotigs and singletons can be seen in Figure 1 and 2, respectively. The isotigs ranged from 19 to 6,450 bp, with an average size of 1,048 bp. The singletons ranged from 50 to 1,081 bp with an average size of 318.6 bp (Table 2). The length of the unigenes ranged from 19 to 6450 bp with an average of 437 bp, and the N50 value was 503 bp, which is greater than that reported in previous studies [39]–[41]. Among the unigenes, 62,841 (64.47%) ranged from 200 to 600 bp, 7,552 unigenes (7.75%) ranged from 601 to 1000 bp and 241 unigenes (0.25%) were larger than 3,200 bp (Figure 3). The results suggest that the Chinese jujube transcriptome sequencing data was effectively assembled, which was further validated by the high proportion of unigenes matched with known protein and the high PCR success rate of the SSR markers developed from the assembled unigenes. A large number of genes are represented in the current combined transcriptome, which will provide an important basis for the mining of genes that are associated with fruit development in Chinese jujube.

**Table 2:** Assembly results of fruit transcriptome of Chinese jujube.

Type	Contig	Isotig	Singleton	Unigene
Total sum	25,805	20,689	76,790	97,479
Total residue	16,093,834	21,682,461	24,462,600	46,145,061
Length range	1–4,622	19–6,450	50–1,081	19–6,450
Average length	623.7	1,048	318.6	473.4
N50	892	1,241	403	503

### Functional annotation of unigenes

**Figure 4:**
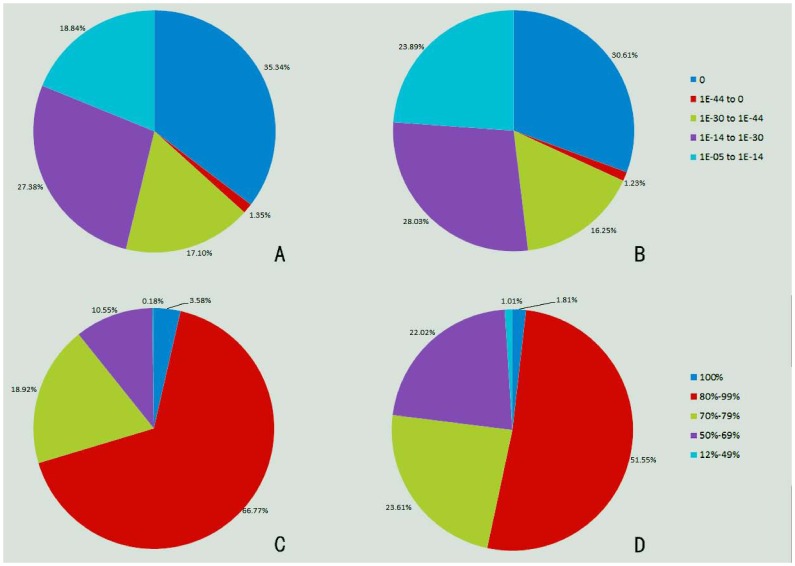
Characterization of the searched assembled unigenes against the nr and SwissProt protein databases. (A) E-value distribution of the BLAST hits for the assembled unigenes with a cutoff of 1e-5 in the Nr database. (B) E-value distribution of the BLAST hits for the assembled unigenes with a cutoff of 1e-5 in the SwissProt database. (C) Similarity distribution of the top BLAST hits for the assembled unigenes with a cutoff of 1e-5 in the nr database. (D) Similarity distribution of the top BLAST hits for the assembled unigenes with a cutoff of 1e-5 in the SwissProt database.

For the functional annotation, we compared all of the unigenes to the NCBI nr database using BLASTx with an E-value cutoff of 1e-5. As a result, of the 97,479 assembled sequences (contigs plus singletons), 52,938 (17,694 contigs and 35,244 singletons) had significant matches (Table S1), representing the putative functional identifications of more than half of the assembled sequences (54.31%) (Table 3). A total of 44,147 unigenes had no significant matches to any known protein and may be considered putative novel transcribed sequences. Because the significance of sequence similarity depends in part upon the length of the query sequence, short sequencing reads that are generated by next-generation sequencing frequently cannot be matched to known genes [38], [42]. Previous studies have shown that 54.9% of these sequencing reads were identified in bamboo [43], 72% in cucumber [44] and 29.7% in Oriental River Prawn [45]. We only achieved half of the number of the assembled unigenes due to the lack of genomic information in this non-model species. To further analyze the BLAST results, the E-value and similarity distributions were calculated (Figure 4). Of the mapped sequences, 18,708 (35.34%) had annotated proteins (<1.0e-50) (Figure 4A), and 37,241 sequences (70.35%) had a BLAST result above the cutoff value and alignment identities greater than 80% (Figure 4A and 4C). These results reflect the high identities of the mapped sequences, suggesting that the sequences have a good assembling quality. The largest number of hits was against *Prunus persica*, followed by *Theobroma cacao, Vitis vinifera, Ricinus communis, Populus trichocarpa, Fragaria vesca subsp. Vesca* (Table S1).

**Table 3:** Summary of function annotation of assembled unigenes.

Database category	Number of unigene hits	Percentage(%)*
NCBI NR	52,938	54.31%
Swiss-prot	29,384	30.14%
GO	33,123	33.98%
Uniref90	52,648	54.01%
COG	9,950	10.21%
KEGG	16,693	17.22%

^*^Proportion of annotated unigenes in total 97,479 assembled unigenes of Chinese jujube (*Ziziphus jujuba* M.).

When mapped with the SwissProt database, 29,384 unigenes obtained significant hits (Table S2), which accounted 30.14% of the total assembled unigenes (Table 3), comparatively less compared with that mapped with the nr database. Similar results were observed in other studies [46], [47]. The functional annotation that was provided by the SwissProt database was considered to be more reliable because it is a high-quality annotated and non-redundant protein sequence database [46]. Of the mapped sequences, 8,993 (30.61%) had annotated proteins (<1.0e-50) (Figure 4B), and 15,678 sequences (53.36%) had a BLAST result above the cutoff value and alignment identities greater than 80% (Figure 4B and 4D). The annotation results suggest that the Roche 454 GS FLX Titanium sequencing project generated a substantial fraction of the Chinese jujube genes in this study.

### COG Classification

**Figure 5:**
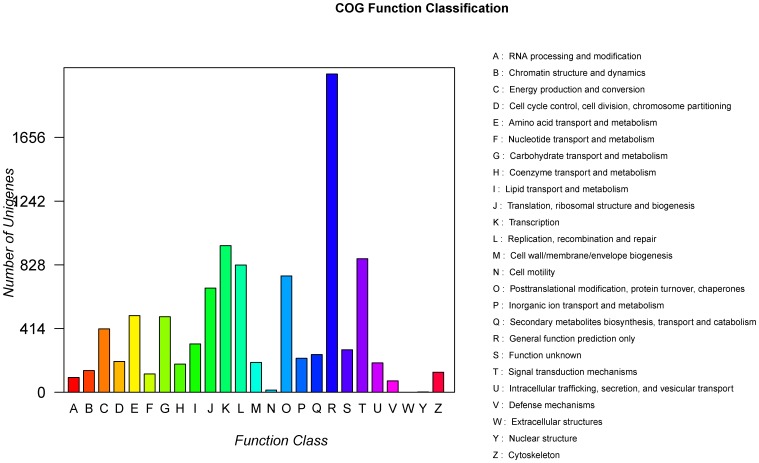
Histogram of the COG classification cluster of Chinese jujube sequences that were deposited in the NCBI database and *de novo*-assembled unigenes. A total of 950 assembled unigenes were annotated and fell into 24 clusters. The Abscissa is the content of the categories of COG, while the ordinate is the number of unigenes.

To further elucidate the functionality of the Chinese jujube transcriptome, the annotated unigenes were categorized into different functional groups based on the **Cluster of Orthologus Groups** (COG) database (Figure 5). Out of the 53,332 annotated unigenes, 9,950 could be classified into 24 COG categories (Table 3). The identity ratio in our study was greater than 1.52% in *Lycoris aurea* [48] and 3.82% in Oriental River Prawn [45] and smaller than 24.42% in rubber tree [49]. Among the aligned COG classifications, the category of general function prediction (2,068, 20.78%) was the largest function group, followed by transcription (953, 9.58%), signal transduction mechanisms (868, 8.72%), replication, recombination and repair (827, 8.31%), posttranslational modification, protein turnover, chaperones (756, 7.60%), translation, ribosomal structure and biogenesis (677, 6.80%) and amino acid transport and metabolism (498, 5.00%) (Table S3). The two categories involving cell motility and nuclear structure consisted of 14 (0.14%) and 2 (0.02%) unigenes, respectively, representing the two smallest COG classifications (Figure 5). In the different functional classes, the number of genes reflected the metabolic or physiological bias under the corresponding environment.

### KEGG Pathway Mapping

**Figure 6:**
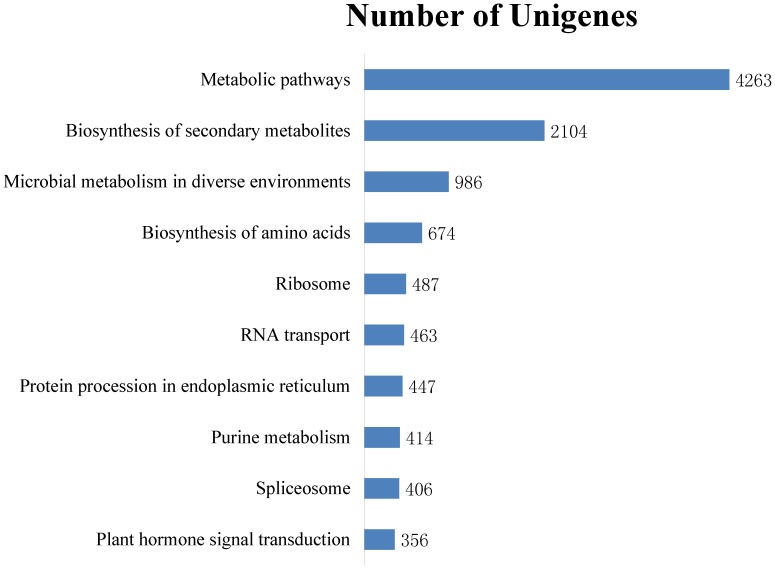
Top ten of unigenes in the Pathway assignment based on the Kyoto Encyclopedia of Genes and Genomes (KEGG).

To explore the active biological pathways in Chinese jujube, a KEGG [50] analysis with an E-value cutoff of 1e-5 was conducted. A total of 53,332 annotated unigenes were found to have significant matches, with 16,693 hits (6,485 isotigs and 10,117 singletons) in the KEGG database assigned to 319 KEGG pathways (Table 3 and Table S4). Previous studies have shown that 21,274 *L. aurea* unigenes, comprising 7,097 contigs and 14,177 singletons, were mapped to 295 KEGG pathways [48] and that 42,126 Oriental River Prawn unique sequences, comprising 39,302 contigs and 2,824 singletons, were mapped to 123 and 90 predicted KEGG pathways, respectively [45]. As shown in Figure 6, 4,263 unigenes were assigned to metabolic pathways, and 2,104 unigenes were clustered into the biosynthesis of secondary metabolites, followed by microbial metabolism in diverse environments (986 unigenes), biosynthesis of amino acids (674 unigenes) and ribosomes (487 unigenes) (Figure 6). Therefore, the metabolic pathways and biosynthesis of secondary metabolites pathways were the two largest pathways, indicating that diversifying metabolic processes are active in Chinese jujube and that a variety of metabolites are synthesized during fruit development. These annotations provide a valuable resource for investigating the specific processes, functions and pathways in fruit development in Chinese jujube.

### Gene Ontology (GO) Classification

**Figure 7:**
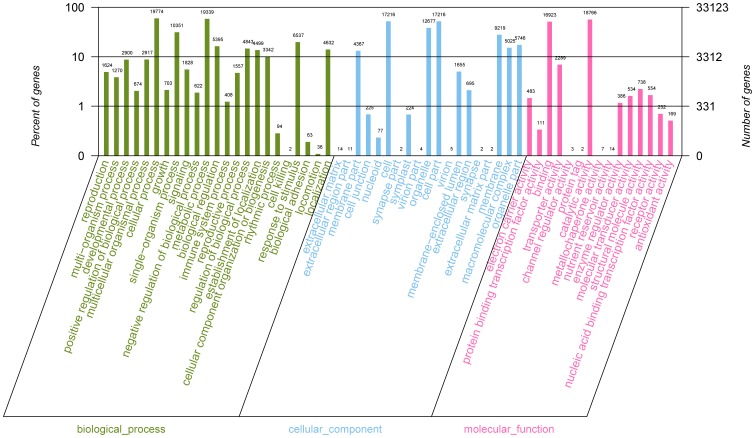
Histogram of GO classifications of assembled Chinese jujube (*Ziziphus jujuba M.*) unigenes. Results are summarized for three main GO categories: biological process, cellular component and molecular function.

The Blast2GO program [51] was used to analyze the GO annotation of the assembled unigenes. The results indicate that 33,123 of the 53,332 annotated unigenes were assigned to one or more GO terms (Table 3). All of the GO terms were categorized into 45 GO function groups, which can be distributed into three main categories: molecular function, biological process and cellular components (Figure 7). In terms of molecular function, the most represented functions were catalytic activity and binding, accounting for 56.7% and 51.1% of the total GO annotations, respectively, followed by channel regulator activity, protein tag, metallochaperone activity and nutrient reservoir activity, whereas the other molecular functions were represented at a much lower scale. Concerning the biological process, most of the represented categories were cellular processes and metabolic processes, with 59.7% and 58.4% of the total GO annotations, respectively. Only a few unigenes were associated with cell killing and locomotion in their biological processes. The cell part and cell each occupied 52.0% of the total GO annotations, followed by the extracellular matrix, extracellular region, synapse, virion, synapse and extracellular matrix (Table S5).

### Genes that are related to the biosynthetic pathway of vitamin C and cAMP

**Figure 8:**
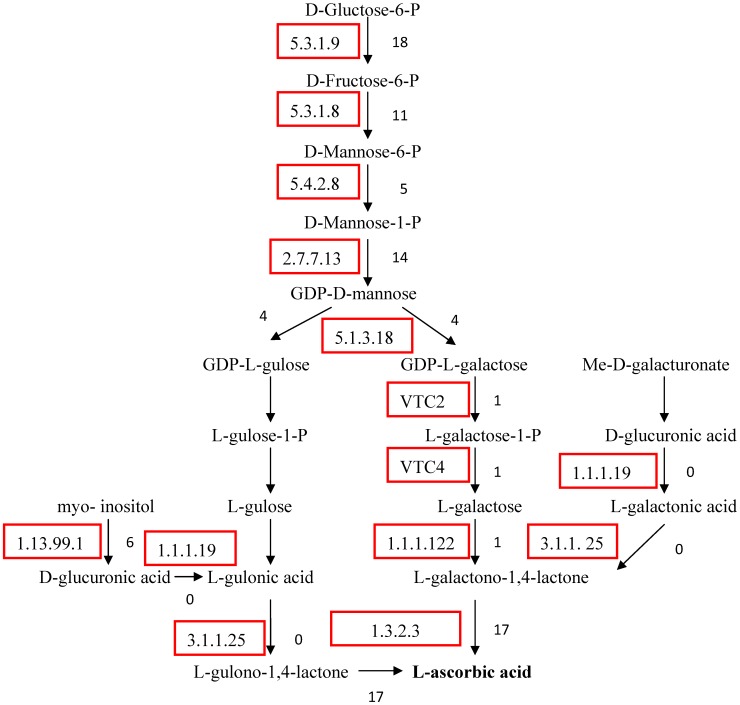
Ascorbic acid biosynthesis pathway in Chinese jujube. The box shows the metabolic enzyme in each step, and the number of unigenes is shown in the right of each step.

Chinese jujube has a high ascorbic acid (AsA, vitamin C) content, which ranged from 166 to 808 mg/100 g among the different cultivars, with an average of 412 mg/100 g [52]. Therefore, it appears that Chinese jujube is a good source of vitamin C in the diet. There are four biosynthetic pathways for AsA in plants, i.e., the Smirnoff-Wheeler (S-W), galactonate, glucose, and myo-inositol pathways [53]–[56], which may be selectively adopted among different plants [46], [54], [57]. The Smirnoff-Wheeler pathway converts D-glucose to L-ascorbate in a 10-step process [58]. In Chinese jujube, the sequences for all nine of the genes in the S-W pathway were assembled; however, the assembly did not occur in the other three pathways (Figure 8). Therefore, we hypothesized that the S-W pathway is the main pathway for the biosynthesis of AsA in Chinese jujube, which is similar to the pathway that was revealed for Chinese bayberry [54]. However, the galactonate pathway was first discovered in strawberry [54], whereas the kiwifruit possesses both the S-W and the myo-inositol pathways [57]. This information will be helpful in understanding the biosynthetic pathway of AsA in Chinese jujube.

Cyclic adenosine monophosphate (cAMP) is a second messenger that has been implicated in many biological processes and has proven effective in the treatment of hypertension, diabetes, cancers, cardiac shock and cardiovascular diseases [59]. Cyong and Hanabusa first extracted cAMP from Chinese jujube [60], and Hanabusa et al. found that the cAMP content of Chinese jujube was 10 times higher than that of other plants [61]. Later, Liu and Wang discovered that the cAMP content was approximately 100–600 nmol/g in various Chinese jujube cultivars [62]. Thus, Chinese jujube is a potential source of cAMP. ATP is catalyzed by adenylyl cyclase to generate cyclic AMP, and cyclic AMP decomposition into AMP is catalyzed by the enzyme 3′,5′-cyclic adenosine monophosphate phosphodiesterase [63]. Among the unigenes, two and four were assembled for 3′,5′-cyclic adenosine monophosphate phosphodiesterase and adenylyl cyclase, respectively. None of the identified unigenes showed significantly different expression between the early and late stages.

### Genes that were differentially expressed in the early versus late stages of fruit development

The Fragments Per Kilobase of transcript per Million mapped reads (FPKM) method [64] is widely used in the expression annotation of RNA-seq data. To obtain a general view of the different expressed genes between the early and late stages in fruit development in Chinese jujube, we used this method to perform a pairwise comparison of the expression abundance between the S1 and S2 libraries. In total, 1,764 unigenes showed significantly different expression. Compared to that of the S2 library, the numbers of upregulated and downregulated genes in the S1 library were 790 and 974, respectively (Table S6 and S7).

**Figure 9:**
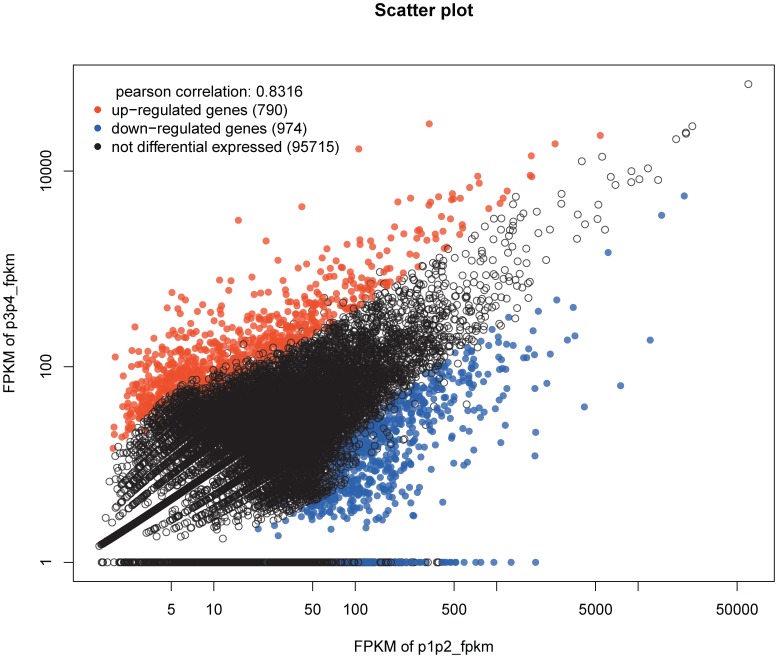
Scatter plot showing the gene expression differences between S1 and S2. The abundance of each gene was normalized as Fragments Per Kilobase of exon model per Million mapped reads (FPKM). The differentially expressed genes are shown in red and blue, while the black indicates genes that were not differentially expressed between S1 and S2.

**Figure 10:**
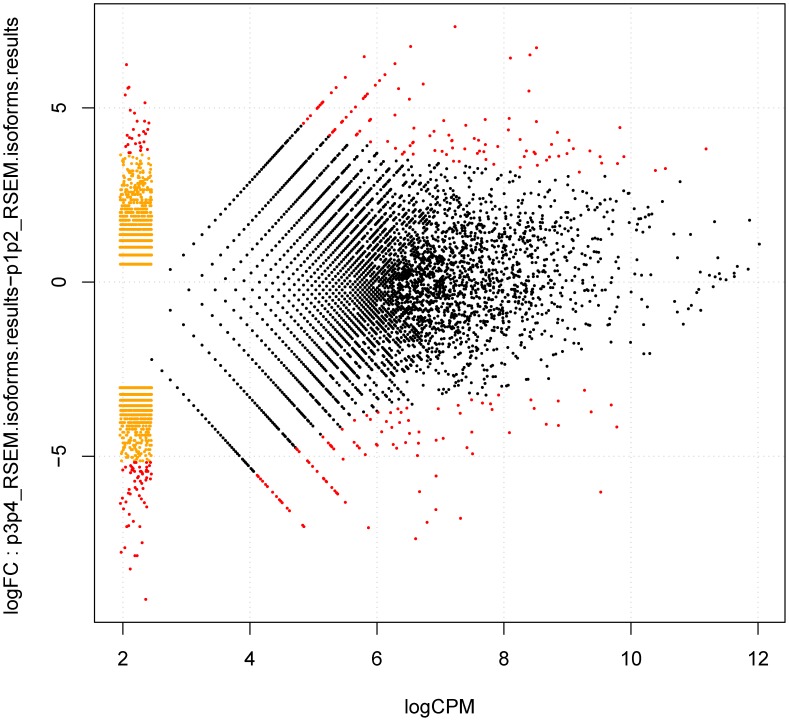
Graphical visualization of significantly differentially expressed genes versus transcripts as designed by the RSEM software. The *y*-axis indicates differences in the logarithms between the expression of the ‘g’ gene of the sample K, K ', while the *x*-axis indicates the average of the logarithm for the expression of the ‘g’ gene of the sample K, K '. log CPM: the average log2-counts-per-million.

A scatter plot and Pearson correlation coefficients showed a positive linear relationship between the two developmental stages (Figure 9), suggesting that the expression patterns of most of the genes were similar and that only a small portion of the genes were expressed differentially. Figure 10 was designed using the RSEM software and further demonstrated the significantly differentially expressed genes versus transcripts using a graphical visualization method. The number of genes upregulated over time was lower than that of the downregulated, differentially expressed genes, indicating that many genes were expressed at lower levels during the late stages compared to their expression during the early stages. For example, it is interesting that isotig16220 and isotig02822 were found to be abundantly expressed during the early stage and were not expressed during the late stages (Table S6). Isotig16220 had a high degree of similarity to the Bowman-Birk inhibitor (BBI) gene, and isotig02822 was annotated as a putative pathogenesis-related thaumatin superfamily protein (PRs). Qu et al. suggested that proteinase inhibitors that were encoded by the BBI gene confer resistance to pathogens or insects in developing seeds, which is an important embryonic defense mechanism [65]. PRs are proteins that are encoded by the plant genome and induced specifically in response to infections by pathogens, such as fungi, bacteria or viruses, or by adverse environmental factors, which function as part of the plant defense system [66]. The results herein may indicate an important role of the BBI gene and PRs during the early stages of fruit development in Chinese jujube.

**Figure 11:**
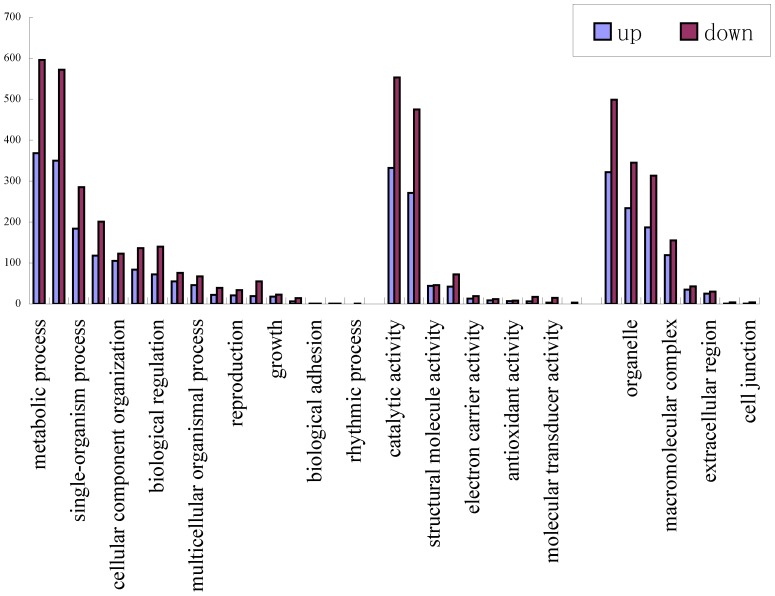
Up-regulated and down-regulated UniGenes expression profiles during Chinese jujube fruit development presented by GO classification.

The numbers and comparisons between the upregulated and downregulated unigenes were summarized in three main functional categories by Blast2GO (Figure 11). Among the differentially expressed genes, those that are involved in metabolic processes, cellular processes, catalytic activity and the cell were notably enriched. A pathway analysis was used to determine the significant pathways involving these differentially expressed genes based on the KEGG pathway database. There were five significant pathways that involved the differentially expressed genes (p < 0.05, Table S8). For example, the unigenes in the flavonoid biosynthesis pathway were expressed at higher levels in the S1 library than in the S2 library, suggesting a decreased accumulation of the related metabolites during the late stages of fruit development.

### Identification of SSR loci in the Chinese jujube fruit transcriptome

Using a MISA analysis, a total of 9,893 sequences were identified containing SSRs from 97,479 unigenes, resulting in a percentage of 10.1%, which is lower than that which was observed in the floral transcriptome of the related species *Z. celata* (17%) [36]. The number of sequences containing more than one SSR locus was 1,628. In total, 11,929 SSRs were identified in the 9,893 sequences, with one SSR detected for every 3.87 kb of the examined sequences (Table 4). In previous studies, one SSR locus for every 3.55 kb (kb/SSR) was detected in *Camellia sinensis* L. [67], 6.22 kb/SSR in the peanut transcriptome [68], 6.69 kb/SSR in the *Epimedium* transcriptome [69] and even 17.56 kb/SSR for *Cymbidium ensifolium* [70]. The frequency of the SSRs in various plant genomes is influenced by the repeat length and the criteria that are used to search the SSRs in database mining [26]. The number of perfect SSRs was 10,633, whereas 1,296 of the SSRs were present in compound formation (Table 4) according to the classification criteria proposed by Weber [71].

**Table 4:** Summary of identifying SSR loci from the unigenes of Chinese jujube.

Category	Numbers
Total number of sequences examined	97,479
Total size of examined sequences (bp)	46,145,061
Total number of identified SSRs	11,929
Number of SSR containing sequences	9,893
Number of sequences containing more than 1 SSR	1,628
Number of SSRs present in compound formation	1,296

**Figure 12:**
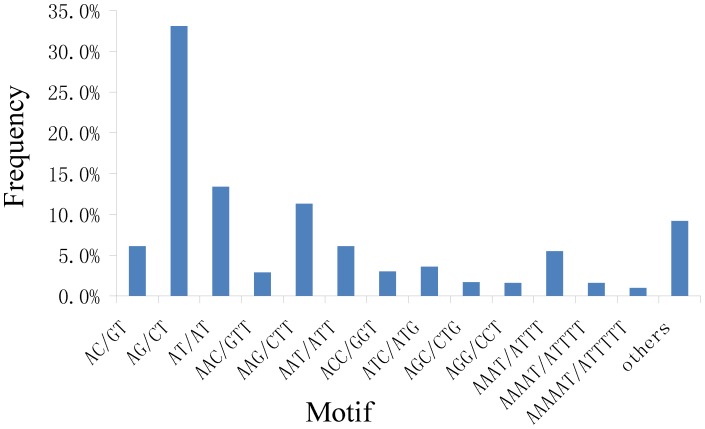
Frequency of classified novel developed SSR repeat types.

The number of SSRs for di-, tri-, tetra-, penta- and hexa-nucleotide repeat types was 6,280, 3,822, 1,150, 356 and 321, respectively. A total of 306 motif sequence types were identified, including 12, 59, 87, 49 and 99 of the di-, tri-, tetra-, penta- and hexa-nucleotide repeat types, respectively (Figure 12 and Table S9 and S10). Among the perfect SSRs, the di-nucleotide (52.64%) repeat type was the most abundant, followed by the tri-nucleotide repeat type (32.04%) (Table 5). A high frequency of di-nucleotide repeats has also been reported in eucalyptus [72] and citrus [73], whereas the tri-nucleotide repeat was the main type in the rice, wheat, barley and *Z. celata* transcriptome sequences [74]. The dinucleotide repeats (AG/CT)n were predominant, representing 62.9% of all of the characterized di-nucleotides, followed by the (AT/AT)n, (AC/GT)n and (CG/CG)n repeats. The (AG/CT)n, (AT/AT)n and (AC/GT)n repeats composed 99.9% of the characterized di-nucleotides. The tri-nucleotide (AAG/CTT)n repeats were the most abundant (35.3%), followed by the (AAT/ATT)n, (ATC/ATG)n, (ACC/GGT)n and (AAC/GTT)n repeats. All of the above tri-nucleotide repeats comprised 83.8% of the characterized tri-nucleotides. For Chinese jujube, the majority of tri-nucleotide repeats ranged from 15 to 30 bp in length (Table S9 and S10), which is consistent with ranges in faba bean, while the most common motif sequence types among the various SSR repeats were different from those of faba bean [75]. A total of 87 tetra-nucleotide repeat motifs were identified, with the most common being AAAT/ATTT (57.0%) and AAAG/CTTT (10.6%). The penta-nucleotide and hexa-nucleotide motifs were comparatively less frequent, together comprising only 5.7% of the total detected SSRs. The dominant pentanucleotide motif was AAAAT/ATTTT (54.8%), and the most common hexanucleotide motif was AAAAAT/ATTTTT (17.8%) (Table S9 and S10). These results indicate that transcriptome sequencing is a good tool for SSR identification in Chinese jujube.

**Table 5:** Distribution of different repeat type classes in fruit transcriptome of Chinese jujube.

Unit size	Number of SSRs	Percent (%)	Frequency (%)
2	6,280	52.64	6.44
3	3,822	32.04	3.92
4	1,150	9.64	1.18
5	356	2.98	0.37
6	321	2.69	0.33
Total	11,929	100	12.24

### Development of novel microsatellite markers and polymorphism testing

A total of 141 primer pairs were specially designed from the sequences with tri-nucleotides repeat types, which were tested for amplification in ‘Dongzao’. Of these primer pairs, 93 amplified repeatedly and reliably, while the other primer pairs produced no bands or too many undesired bands and were, therefore, excluded from further analysis. In this study, a high success ratio of amplification efficiency (71.6%) was obtained compared to similar studies and was similar to that which was observed for tri-nucleotide repeats in *Camellia sinensis* L. [67].

**Figure 13:**
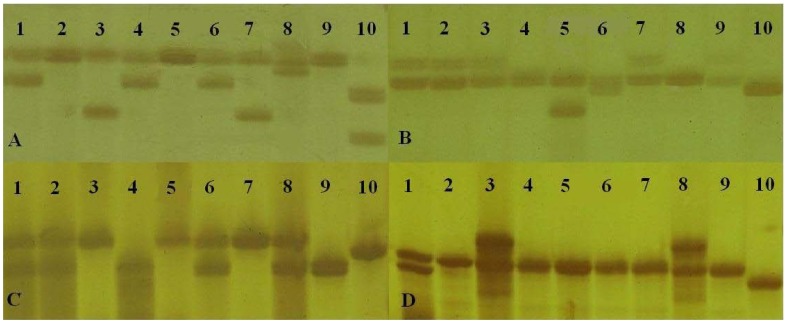
Silver stained polyacrylamide gels of four example SSR loci showed microsatellite polymorphisms in Chinese jujube and related species. The SSR loci BFU2038, BFU2113, BFU2134 and BFU2143 are corresponding to A, B, C and D, respectively. Lane1, ‘Hupingzao’; Lane2, ‘Yuanling’; Lane3, ‘Dongzao’; Lane4, ‘Junzao’; Lane5, ‘Jidanzao’; Lane6, ‘Mayizao’; Lane7, ‘Jinsixiaozao’; Lane8, ‘Huizao’; Lane9, ‘Anonymous’; Lane10, ‘Wanglang’.

To investigate the transferability and to validate the level of polymorphism for the newly designed SSR markers, all of the successful primer pairs were tested on ten *Ziziphus* accessions, including eight accessions of *Z. jujuba*, one accession of *Z. acidojujuba* and one accession of *Z. mauritiana* Lam (Table 6). As a result, polymorphism of four example SSR loci were demonstrated in Figure 13. Only 3 loci (BFU2101, BFU2112, and BFU2114) could not amplify in *Z. mauritiana Lam*, whereas all of the loci could amplify successfully for *Z. acidojujuba*. Thus, these results indicate that the novel SSR primers have good transferability to other cultivars of *Z. jujuba* and even to the closely related species *Z. jujuba* and *Z. acidojujuba.* Furthermore, 71 markers (76.3%, Table S11) showed polymorphisms in the analyzed accessions. The marker polymorphisms observed in this study are higher than those of similar studies; for example, 47.5% was observed in *Lens culinaris* Medik. [76], and even 12.9% was detected in pigeonpea (*Cajanus cajan* (L.) Millspaugh) [77]. *Z. mauritiana* Lam is comparatively distant to Chinese jujube, and the polymorphic rate decreased to 49.5% when this species was excluded from the analysis. Generally, due to the conserved nature of EST-SSR, fewer polymorphisms were detected for EST-SSRs than for genomic SSRs [78]. However, a high polymorphic rate was also observed in some of the EST-SSR studies [79], [80]. The *Ziziphus* accessions that were used in this study were selected based on previous studies; the high degree of divergence among these accessions may be the reason for the revealed high polymorphic rate. The novel primers developed herein may represent highly conserved genes with some important biological/cellular/molecular functions, which would be useful for studying the functional diversity of Chinese jujube cultivars and for performing association mapping studies.

**Table 6:** List of the *Ziziphus* accessions used in this study.

NO.	Cultivar name	Species name
1	‘Hupingzao’	*Z. jujuba*
2	‘Yuanling’	*Z. jujuba*
3	‘Dongzao’	*Z. jujuba*
4	‘Junzao’	*Z. jujuba*
5	‘Jidanzao’	*Z. jujuba*
6	‘Mayizao’	*Z. jujuba*
7	‘Jinsixiaozao’	*Z. jujuba*
8	‘Huizao’	*Z. jujuba*
9	‘Anonymous’	*Z. acidojujuba*
10	‘Wanglang’	*Z. mauritiana* Lam.

In this study, only tri-nucleotide repeats were adopted to develop the SSR loci (Table S12). Di-nucleotide repeats have the disadvantage of high amounts of stuttering, which require very accurate and reliable protocols for allele separation and identification to avoid allele mis-calling, while tri-nucleotide repeats and other longer core motifs can be more integral and greatly reduce arbitrary binning decisions. Long nucleotide repeats for SSRs were selected for fingerprinting, as previously adopted in many species, including humans and grape [81]. Cipriani et al. proposed a set of SSR markers with long core repeats for grape genotyping [81]. Using capillary electrophoresis, the tri-nucleotide repeats were found to be more robust than the di-nucleotide repeats; due to the lower number of stuttering bands, di-nucleotide repeats exhibited a lower separation of neighboring alleles and a higher amount of stuttering bands, which make the interpretation of electropherograms and the calling of true alleles less reliable [82]–[85]. The tri-nucleotide core repeats are less frequent in most plant genomes [86]–[88]; therefore, the development of tri-nucleotide repeats was more convenient when taking advantage of high-throughput transcriptome sequencing.

A total of 194 alleles were detected using 71 polymorphic primer pairs, ranging from 2 to 6, with an average of 2.75. Among these primer pairs, BFU2055 produced a maximum of 6 alleles. The effective number of alleles per locus, which reflects the evenness of the allelic frequencies, ranged from 1.1 to 3.7, with an average of 1.8. The polymorphic information content (PIC) value for the primer pairs ranged from 0.09 to 0.63 with an average value of 0.34 (Table S11). Only 13 primer pairs were highly polymorphic with a PIC greater than 0.5 according to the criterion suggested by Botstein et al. [89]. The observed and expected heterozygosities ranged from 0.1 to 0.9 and 0.1 to 0.77, respectively.

## Conclusions

In this study, we present the sequencing, *de novo* assembly and annotation of the Chinese jujube fruit transcriptome. In total, 1,124,197 high-quality reads were obtained, and 97,479 unigenes were assembled. Furthermore, the differentially expressed genes between two different developmental stages of the Chinese jujube fruit were revealed. The biosynthesis pathways of ascorbic acid and cAMP were involved in fruit development in the Chinese jujube. Furthermore, a set of genic SSR primer pairs were developed that will help in genetic and breeding studies of Chinese jujube and related species. These results further highlight the efficient identification of SSRs from the Chinese jujube transcriptome, indicating that transcriptome sequencing is an effective tool for primer development.

## Materials and Methods

### Plant material

*Z. jujuba* ‘Dongzao’ (Dongzao), a cultivar with excellent fresh fruit quality, was grown in fields of the Chinese Jujube Experimental Station (CJES) of Beijing Forestry University in Cang County, Hebei Province, China, under normal cultivation conditions. A fruit growth curve of ‘Dongzao’ was developed based on the fruit weight as showed in Figure S1. Based on the curve, the fruit samples were collected 19 days after flowering (DAF, P1), 25 DAF (P2), 63 DAF (P3) and 85 DAF (P4). The fruit flesh and skin from three fruits were mixed, placed immediately into liquid nitrogen and stored at −80°C until use.

Seven cultivars of *Z. jujuba* and one accession from *Z. mauritiana* Lam and *Z. acidojujuba* (Table 6) were selected for the SSR polymorphism and transferability tests. The leaves from nine accessions were also collected from CJES. The leaves were immediately frozen in liquid nitrogen and stored at −80°C until use. The DNA was isolated using a modified CTAB (Cetyltrimethyl Ammonium Bromide) method [90]. The plant material employed in this study was collected from our own germplasm repository, thus no specific permits were required for the collection. In addition, the field study did not involve endangered or protected species.

### cDNA library creation, 454 sequencing and assembly

The total RNA of the ‘Dongzao’ (Dongzao) fruit tissue was extracted using TRIzol reagent (Sigma-Aldrich, Saint Louis, USA) following the manufacturer's instructions, and the RNA was purified using RNeasy plant mini kit (Qiagen, Hilden, Germany). The total RNA from P1 and P2 and also from P3 and P4 were pooled in equal amounts. Thus, two cDNA libraries (S1 and S2) were constructed for transcriptome sequencing using the SMART cDNA Construction Kit (Clontech, USA). S1 represents the early fruit development stage, whereas S2 represents the late development stage. Subsequently, a half-plate 454 sequencing run with GSFLX Titanium was performed for each library at Meiji Biotechnology Co., Ltd (China) following the manufacturer's instructions.

The raw sequencing reads were first filtered by removing the low-quality reads and the adaptor sequences using the EMBOSS software package [91]. The Newbler software (Roche version 2.3) was used to *de novo* assemble the reads into unigenes. The reads from S1 and S2 were first assembled separately; afterwards, the reads of S1 and S2 were assembled together to obtain a complete reference transcriptome.

### Functional characterization and annotation

The BLAST program was used to align the resulting unigene sequences against the GenBank non-redundant database with a cutoff E-value of 1e-5. The BLASTx search tool was also used to align the unigenes with the SwissProt, KEGG and COG databases (released in October 2013) with a cutoff E-value of 1e-6. The proteins with the highest sequence similarity were retrieved for further analyses. Gene Ontology (GO) annotations were performed to predict the molecular function, biological processes and cellular component terms using Blast2GO [51]. Annotations against the Kyoto Encyclopedia of Genes and Genomes (KEGG) database (http://www.genome.jp/kegg/) were performed to determine the links between enzymes, integrating together the genomic, chemical and network information using the Blast2GO program [51]. All of the mapped sequences were annotated to the KEGG database to obtain the enzyme commission (EC) number. The EC was then mapped to the KEGG Pathway to obtain the KEGG Pathway Maps [50]. The KOBAS 2.0 (http://kobas.cbi.pku.edu.cn/home.do) software was employed to identify the enriched pathways. The hypergeometric distribution was used for the p-value calculation. The false discovery rate (FDR) was calculated using the Benjamini and Hochberg method [92], and for the other parameters, the defaults were used.

### Identification of the differentially expressed genes

The differentially expressed genes between the S1 and S2 stages were identified using edgeR (http://www.bioconductor.org/packages/release/bioc/html/edgeR.html) and RSEM (http://deweylab.biostat.wisc.edu/rsem/). In this study, the differentially expressed unigenes between each of the two samples were screened with the threshold FDR<0.05 and the absolute value of |logFC|>1. The gene expression level was estimated by the number of clean reads that were located in the genomic regions. The number of mapped clean reads for each unigene was counted and then normalized into the FPKM value (Fragments Per Kb per Million reads) [93]; subsequently, a final visualization analysis was performed. Furthermore, the GO classification was compared between the upregulated and downregulated unigenes using Blast2GO [51].

### SSR loci identification and primer pair design

The MISA program (http://pgrc.ipk-gatersleben.de/misa/) was used to identify the SSR loci. The minimum repeat number was set to six for the dinucleotides and five for the tri-, tetra-, penta- and hexa-nucleotides. Primer premier 5.0 (PREMIER Biosoft International, CA, USA) was used to design the primer pairs flanking the SSR loci with perfect tri-nucleotide motifs using the following parameters: (1) the primers were 18-34 nucleotides long; (2) the primers were devoid of secondary structure or consecutive tracts of a single nucleotide; (3) only G or C was allowed at the 3′ end of the primer; (4) the GC content was approximately 50% and the Tm value was approximately 60°C and (5) the primer designed area was limited to the middle region, with 30 bp being removed from the ends of the contig sequence. The sequences that provided no useful primer pairs were eliminated from further analysis.

### SSR characterization and marker assessment

The designed primers were first tested for amplification using ‘Dongzao’ (Dongzao) and for polymorphism and transferability using ten *Ziziphus* accessions. The PCR was performed in a total volume of 25 µl containing 1.5 mM MgCl_2_, 0.2 mM dNTP, 0.5 µM each primer, 1× PCR buffer, 0.3 µl (2.5 units) Taq polymerase (Takara Biotechnology, Dalian, China), and 10 ng genomic DNA. The thermal cycling program consisted of pre-denaturation at 94°C for 10 min, 35 cycles at 94°C for 45 s, 54°C–56°C for 45 s and 72°C for 45 s, followed by a final extension at 72°C for 3 min. The PCR products were separated on 6% denaturing polyacrylamide gels and visualized by silver nitrate staining following the protocol that was described by Pang et al. [94]. The polymorphism information content (PIC) value for each primer pair was calculated by PopGen 32 Version 1.31[95].

## Supporting Information

Figure S1 **Chinese jujube fruit growth and developmental stages considered for library construction.** Chinese jujube fruit (cv ‘Dongzao’) growth curve expressed as fresh weight (g) accumulation. For the two libraries S1 and S2, the fruit from samples collected at stages P1, P2 and P3, P4 were combined, respectively.
(TIF)

Table S1 **Assembled unigenes matched with NR database.**
(XLS)

Table S2 **Assembled unigenes matched with Swissprot database.**
(XLS)

Table S3 **Assembled unigenes matched with COG database.**
(RAR)

Table S4 **Assembled unigenes matched with KEGG database.**
(XLS)

Table S5 **Assembled unigenes matched with GO database.**
(XLS)

Table S6 **List of up-regulated unigenes by compared with S2 stage.**
(XLS)

Table S7 **List of down-regulated unigenes by compared with S2 stage.**
(XLS)

Table S8 **List of KEGG enrichment pathway based on different genes.**
(XLS)

Table S9 **Results of microsatellite search for all Isotigs.**
(TXT)

Table S10 **Results of microsatellite search for all Singletons.**
(TXT)

Table S11 **Characteristics of 93 SSR markers in ten** ***Ziziphus*** **accessions.**
(DOC)

Table S12 **List of contigs containing tri-nucletide core repeat type SSRs.**
(TXT)
