# Supplementary material for: De Novo Assembly and Characterization of the Fruit Transcriptome of Chinese Jujube (Ziziphus jujuba Mill.) Using 454 Pyrosequencing and the Development of Novel Tri-Nucleotide SSR Markers
Source: PLoS One. 2014 Sep 3;9(9):e106438. doi: 10.1371/journal.pone.0106438 (PMC4153635; doi:10.1371/journal.pone.0106438)
Supplement: Table S11 — Characteristics of 93 SSR markers in ten Ziziphus accessions. (DOC) [file pone.0106438.s012.doc]

Additional File 10: Characteristics of 93 SSR markers in ten *Ziziphus* accessions

| **Primer** | **Repeat motif** | **PIC** | **Forward Primer (5’–3’)** | **Reverse Primer (5’- 3’)** | **Ta（℃）** | **size(bp)** |
| --- | --- | --- | --- | --- | --- | --- |
| **BFU2021** | **(TTC)5** | **0.09** | **GGCGAACATAACCGAGACGAG** | **GCGATATGATGGCAAACACG** | **56** | **224** |
| **BFU2023** | **(TGC)5** | **0.44** | **GCTGAGGGTACATCATCATCTG** | **GGAGGGAGAGAAGCTAGACAAAG** | **58** | **227** |
| **BFU2024** | **(CGA)7** | **0.41** | **GGTGCGACATTGATAACATCTTTAC** | **ACCGTGTATTCATGTCTTGTTTAGG** | **56** | **181** |
| **BFU2026** | **(TGT)6** | **0.16** | **CATGGAATCGTCGTGGGTC** | **AGAAAGGAAAACGGGTCGG** | **56** | **218** |
| **BFU2027** | **(TTG)6** | **0.16** | **GGCAGAGTAGTTATCGAACCAGAAG** | **GGAAGAGGAAACGCCAAAATC** | **57** | **250** |
| **BFU2028** | **(AAG)8** | **0.24** | **GCTTCGGACTAAAGAAAAGAGG** | **CCGGAGTTTAAGAAACCAAACC** | **56** | **227** |
| **BFU2029** | **(CCA)5** | **0.16** | **CTGTTCAACGAGAACATGAAGC** | **GCGGAAGAAATGGACAGATAAC** | **57** | **221** |
| **BFU2033** | **(GGT)14** | **0.22** | **CCATCGTTGACTGTGGCTTTG** | **TTCCCGCTTCTTCTCCTTCC** | **58** | **195** |
| **BFU2036** | **(GTT)5** | **0.16** | **GAGATTGAGTGACAAGACGAGGC** | **GGGGAAGGGGTTGGTTTC** | **56** | **207** |
| **BFU2037** | **(GCT)7** | **0.18** | **AAAATGGGATGGGGATCATAGG** | **AGGCTGAGGAAGAGGAATAGGC** | **58** | **118** |
| **BFU2038** | **(AAG)5** | **0.48** | **AAAGCACGTCAAAACCAGAAAG** | **GCCTGAACTTTGAAGGAAGAAGTAC** | **56** | **182** |
| **BFU2040** | **(ACA)9** | **0.35** | **CTCGGGACGGAGATACTGATC** | **ACTTGAGGACGACGTTGTGG** | **56** | **205** |
| **BFU2042** | **(CCA)7** | **0.63** | **AAACCTCCTTGGAACTTTCGG** | **CTTTTGGACAGCGTTTTGAGTG** | **57** | **213** |
| **BFU2043** | **(GAA)7** | **0.4** | **GATCTCCCTCCCTCTGTTTCTG** | **CTATTCCACATAGAGTAGCCGTTTG** | **57** | **121** |
| **BFU2044** | **(CTT)8** | **0.09** | **TGCACTAGAGCCGAGCATTTG** | **GATCCGTTTCGACTCTTGACC** | **57** | **175** |
| **BFU2045** | **(TCT)5** | **0.16** | **TCCGATTCCGAATCCAAGC** | **TCCAGCAGAAGCAACGACAAC** | **58** | **180** |
| **BFU2048** | **(GGT)9** | **0.44** | **GGCAACCAATCTACACCAAACC** | **TCCACTGCGAGAATCCTTCG** | **58** | **230** |
| **BFU2049** | **(TGT)8** | **0.5** | **ATGCGGTGTTGTTGGTTGAAG** | **CATAACTTCGGGTGTTCCTTCTC** | **57** | **170** |
| **BFU2051** | **(GCC)6** | **0.5** | **CCGGTGGTACAAACAACTCTTG** | **TGGATTCGTCTTCCTCGTCAG** | **55** | **159** |
| **BFU2052** | **(GAA)5** | **0.16** | **TCGAGCGAGTGAGTAGAGGGATC** | **CACGAGGGTTAAGTCTCAGATGC** | **56** | **219** |
| **BFU2054** | **(GCC)7** | **0.37** | **CCTTATGTACCAGTACCGAGCC** | **CAGCATCCAAATCCACCAATC** | **55** | **235** |
| **BFU2055** | **(CAT)7** | **0.69** | **CCTTAAATGCTGGACGACAACC** | **AAGCAGCGATGACGAAGAGC** | **55** | **137** |
| **BFU2056** | **(CTG)7** | **0.37** | **CGGGTGTAAAGAATCCAAAGG** | **TGCAAGGCACTGACAAATCG** | **55** | **214** |
| **BFU2058** | **(CCA)6** | **0.16** | **GCGGAGGGATTGAGTTGTG** | **GAATGAAGACGAAGACGACTGG** | **54** | **147** |
| **BFU2059** | **(GTG)6** | **0.44** | **CCCTCAGTTTCCTCTTCCATAAG** | **GAGCATCGTTGATACGCTTCC** | **55** | **155** |
| **BFU2061** | **(AGC)5** | **0.09** | **GCTGCTGAGGCATTGCTTTC** | **TTCCGACGATTAGGGGCTTG** | **56** | **237** |
| **BFU2063** | **(TGC)6** | **0.44** | **TTTGCGGGATTTTCCTGG** | **GGCTTTATCTGCGTGGCTTC** | **55** | **110** |
| **BFU2065** | **(TTA)6** | **0.49** | **GAGTAATGATCCGACAGCTCCTG** | **ATAAACCCACCTAACCGCTTTC** | **55** | **153** |
| **BFU2068** | **(CAA)7** | **0.5** | **CCTTCCGATTCCGAACAATG** | **TTTGGTTCCTCGCTAACCTTC** | **55** | **121** |
| **BFU2069** | **(TGC)6** | **0.35** | **TCTTTGGCTCGGAAGGTGG** | **ATGCAGACATACGAGCAGGGTC** | **56** | **161** |
| **BFU2072** | **(CAG)6** | **0.35** | **GGCTTCCGACCAAAACGATG** | **CCTGATACTCCTCCGAGTCAATG** | **56** | **188** |
| **BFU2076** | **(GTG)5** | **0.16** | **AGGCGGTGTTGATTATGATGG** | **GGTTCCGGCGATATGCTAAAC** | **55** | **159** |
| **BFU2077** | **(ACA)10** | **0.55** | **CAACTCCAGAGCCATCGTCAC** | **TTGCGTTCTCCACGGTTTTC** | **55** | **155** |
| **BFU2081** | **(CAA)5** | **0.4** | **ACCCCTCAAACTGTCAACGC** | **TCACAGCCATATCCGAGAACC** | **55** | **199** |
| **BFU2085** | **(CAC)10** | **0.09** | **ACTGGAGATGGCAATGGAGG** | **TCAGCCCACGTATCAGAAGC** | **54** | **236** |
| **BFU2086** | **(AGA)5** | **0.4** | **ATGTCGTCCCCGTTGTTAGC** | **AGGTGAGGGCGATGAGATTG** | **55** | **199** |
| **BFU2087** | **(ATC)5** | **0.18** | **TGTGAAGGTGGCCGATACG** | **AGATACCACCAACCCCAGACC** | **55** | **195** |
| **BFU2089** | **(GGA)6** | **0.09** | **GCCGTGCCAACATAAAACTACG** | **GGTTCTCCAACCTTCCACATTC** | **56** | **150** |
| **BFU2092** | **(CGG)5** | **0.16** | **CGAGCCCTTGCGTTATCATC** | **ACAAGCACCAAGTCCAACGTC** | **55** | **134** |
| **BFU2093** | **(ATG)5** | **0.18** | **CACAACTTGACTGGAACCATTAGG** | **TATGCTAGGTTCTTGACGGCTAAG** | **55** | **195** |
| **BFU2094** | **(GCT)5** | **0.16** | **CCTCAACAACGGTAGCCATG** | **CTGTAGCACTGACTTCGTGAGATG** | **55** | **245** |
| **BFU2095** | **(GAA)5** | **0.5** | **TGGGACTAAGGTTCTAATGGCG** | **CTTGCTCAGTAATGGGGTTTTG** | **55** | **121** |
| **BFU2096** | **(TCT)5** | **0.53** | **CCGTTCCTAACAGTCCTAATGC** | **ATGTGGGGTTGGGGTAGATG** | **54** | **241** |
| **BFU2097** | **(GAG)5** | **0.16** | **ACCGAAGCCACAAAATTAGGAG** | **TCTGGGCAGCAAACATGGAG** | **56** | **245** |
| **BFU2098** | **(ACC)5** | **0.16** | **TGCTTTGACTTGGTGGATGG** | **GATTAGAGGATGAGATTGGTGGC** | **54** | **181** |
| **BFU2099** | **(ATC)7** | **0.48** | **GAGTGAGCAAAAGCCTAAAGAAGC** | **TATCCAAAAGCACCGGGATG** | **56** | **181** |
| **BFU2101** | **(CCA)6** | **0.44** | **GATAACAACGAAAGAGGCATGG** | **CATAGATGACGGAGTTGAGAATGG** | **55** | **176** |
| **BFU2102** | **(TGA)6** | **0.16** | **CCAGACGAAGAAACAGTGCG** | **CCCAAACAGTTTCAGACCCTC** | **54** | **196** |
| **BFU2105** | **(TCT)5** | **0.3** | **TTCAGAAGCTCTGCGGACAAG** | **GAAACCCTAATCACAGCCATCAG** | **55** | **224** |
| **BFU2108** | **(TTC)5** | **0.5** | **CAAGGGATTGGCGTGACTG** | **AGACGCAGAAGACCCAGAAGTG** | **55** | **230** |
| **BFU2111** | **(CAT)8** | **0.37** | **GCGTACACTCGAAGCACAAGTTAG** | **GAGAGGGCATGGTTTCATCAAG** | **56** | **179** |
| **BFU2112** | **(AAT)5** | **0.38** | **CGCCAATTAAAGCCTGTCG** | **TTCCTCGTGGACTCGTCTCG** | **55** | **138** |
| **BFU2113** | **(CAC)7** | **0.51** | **TTTGAGATGCCGAGGCGTAC** | **GGGAATCCGATCCATTTTGC** | **56** | **244** |
| **BFU2114** | **(ATC)6** | **0.57** | **AACCATACAAGTCCGAAAAGGC** | **GGAAACTGCTACAAATGTGACGC** | **56** | **183** |
| **BFU2115** | **(ACC)7** | **0.44** | **CTTCAAGAAACCCTCTGCTGC** | **CACAAACTGGGCATACGACAC** | **55** | **137** |
| **BFU2119** | **(TCT)5** | **0.16** | **TTTCGAGGTCGTCTTGGTAATG** | **CGTACAAGGGTGAAAGTGAGAATG** | **55** | **202** |
| **BFU2120** | **(AGG)7** | **0.37** | **GATCCAAATCTTGCGGTCTCG** | **GGGGTGGACATGGGGTTATC** | **56** | **205** |
| **BFU2123** | **(TGC)6** | **0.16** | **TTTGTATCTCCCAAGGGTCCG** | **AAACTGCCGGGATGTTATCG** | **56** | **214** |
| **BFU2124** | **(TCC)5** | **0.35** | **TTAGTAGGCCAACCGATCTCC** | **TTATCCTTCGCATCCGCTC** | **54** | **227** |
| **BFU2126** | **(CCA)6** | **0.5** | **CGACGTACTATAATCATGGGAGC** | **CGTTGTTTGCGGAAAGAGG** | **54** | **168** |
| **BFU2128** | **(GTT)7** | **0.5** | **CCTGCTGTTTCTCCATTGCTATAC** | **ATTCCCCTTTTGAGGCCAAC** | **55** | **165** |
| **BFU2129** | **(TCC)5** | **0.49** | **GGCGTATGATCTAAAATGGCC** | **CCCGCTTTCGATGTTGTATG** | **54** | **182** |
| **BFU2130** | **(GAT)5** | **0.49** | **CGAGGAGCATCAAACACCAAG** | **TTGGGAAGCAGTGAAAGAACG** | **55** | **227** |
| **BFU2131** | **(ATC)7** | **0.16** | **CTGCTGTTACCACGTTCCTTTC** | **ATCGCTCCCAACATTTCAGG** | **54** | **249** |
| **BFU2134** | **(TGC)7** | **0.49** | **GAGCCGACACCAAACTAAACG** | **TGAGATCCAGTTCTATAACCCACC** | **55** | **218** |
| **BFU2135** | **(TCT)6** | **0.09** | **AGACTGTGAAGTGTCGCAGGAG** | **AAGACGACGAAGACTTGGAGC** | **54** | **228** |
| **BFU2137** | **(TTC)5** | **0.38** | **CCTTTGACGATCCCTTTTCC** | **GAGGCTGTTTGAGGTTTGTGG** | **54** | **166** |
| **BFU2139** | **(ATC)8** | **0.31** | **TGTTCCATTCTTCGATGCTCAC** | **CCTGCAAAAGTCCATAGTCAGG** | **55** | **198** |
| **BFU2140** | **(GAA)5** | **0.09** | **ATACGACGGTCGGTATCAACG** | **TCTCACAGCCACGCCATTTC** | **56** | **244** |
| **BFU2143** | **(TCT)8** | **0.37** | **TAATCAGGCAAATCCTGGAAGC** | **CCGTTTAGGATGATCGAGAACC** | **55** | **194** |
| **BFU2144** | **(TGG)5** | **0.16** | **GAAAAGAGCGTCGAACAGCG** | **GTTTGGGAACATGAGGAAAGG** | **55** | **190** |
| **BFU2010** | **(ATT)5** | **0** | **TCGCGTCCTAAGATGCTG** | **CGATCAAGGCCATTCTCC** | **58** | **365** |
| **BFU2011** | **(ACA)4** | **0** | **CAGAGCACAGAGCAAGGC** | **TTGCGAGACAGCACAGATGAGC** | **56** | **195** |
| **BFU2015** | **(AGT)5** | **0** | **GCAGCGATTATCTGACGG** | **ATGCAGGAAGCGACACGA** | **54** | **192** |
| **BFU2020** | **(CCT)5** | **0** | **ATTATGGTTCCAGGCTTGTCC** | **AGAAGAGTATGGGCGTTTTGAG** | **55** | **239** |
| **BFU2025** | **(GGT)5** | **0** | **GTGTTGGGAATAACCTCGATTC** | **CCTTCTGATCTTGAACCCTAACC** | **56** | **249** |
| **BFU2030** | **(TGC)5** | **0** | **CACCCTTTCATCCTGCCTATG** | **ATTGCTTCCTGTGACCTCTGC** | **56** | **152** |
| **BFU2032** | **(TTG)5** | **0** | **TCCGAATCACCTCCTTCATC** | **CATTGTTCCTCCTCCACCAC** | **57** | **230** |
| **BFU2034** | **(GAA)8** | **0** | **AACCAACCACCTCCTCAACC** | **CGCACCTTCTTCTTCTTCCAG** | **56** | **123** |
| **BFU2039** | **(ATC)8** | **0** | **TTTTGACCCCGAGAACAGCC** | **TATGGGCAGGGCAGAAACAG** | **57** | **156** |
| **BFU2046** | **(TGG)5** | **0** | **GACGCTGATTCTCCAATCGTAAG** | **AACCAAACGCAACCAGGTCC** | **58** | **218** |
| **BFU2053** | **(GGT)5** | **0** | **TCGGGTAAGAGTACAAGAAACGG** | **CTTCTTCCGCAACCTCCAAG** | **55** | **155** |
| **BFU2057** | **(TTC)6** | **0** | **CCATGAGACCATTGGGAAGG** | **CAGGATGGTTCTGAAGAGGGAG** | **55** | **146** |
| **BFU2071** | **(GCT)5** | **0** | **GCGAGGAATGTAATCTGGGTC** | **CATCAAGTAGCACAACTGGAAGAG** | **54** | **230** |
| **BFU2074** | **(GGT)5** | **0** | **GACGATGGGCAACTGGAGTATAG** | **CGATGAAGAAGAAGCCGAGAAG** | **56** | **181** |
| **BFU2078** | **(AAG)6** | **0** | **AAAAGCGTCAAGAAACCGTCAG** | **GCTTCAGCTCCCATTTTCTCAC** | **56** | **190** |
| **BFU2084** | **(ATT)7** | **0** | **GCCAAGAAACAACGGACAATTC** | **CACAGTTCGCCAGAGGGTTATAC** | **56** | **247** |
| **BFU2090** | **(TTG)5** | **0** | **TTCCTTCTCGCACTTGACCTC** | **CGCAACCACCATCACTATTACC** | **55** | **176** |
| **BFU2103** | **(CAA)5** | **0** | **GAAGGCGTTGTGCATTGATC** | **CGAGTTGTGGGTTGTGGAAG** | **54** | **208** |
| **BFU2109** | **(GTG)5** | **0** | **TAATCCTTCTGTACTCGTCGTCAC** | **CAAATCATCCTCCTTCTTCAACC** | **54** | **230** |
| **BFU2122** | **(TCC)5** | **0** | **CTCCTAACGACGAACCGAATC** | **AGTTGTCGAAGGGCAAGGAC** | **54** | **242** |
| **BFU2125** | **(TTC)5** | **0** | **TTACGCCCCTCATCCTCTTC** | **CAAGCATGGCAAACCTAACG** | **54** | **183** |
| **BFU2133** | **(CAA)5** | **0** | **AGCGAGGCATTTGGACGATC** | **CCACCTGTATAAGCAATCACGC** | **56** | **214** |
